# Keyframe image processing of semantic 3D point clouds based on deep learning

**DOI:** 10.3389/fnbot.2022.988024

**Published:** 2023-01-18

**Authors:** Junxian Wang, Wei Lv, Zhouya Wang, Xiaolong Zhang, Meixuan Jiang, Junhan Gao, Shangwen Chen

**Affiliations:** ^1^Alibaba Cloud Big Data Application College, Zhuhai College of Science and Technology, Zhuhai, China; ^2^Department of English, Faculty of Arts and Humanities, University of Macau, Zhuhai, China

**Keywords:** deep learning, 3D point cloud, keyframe, image processing, U-Net

## Abstract

With the rapid development of web technologies and the popularity of smartphones, users are uploading and sharing a large number of images every day. Therefore, it is a very important issue nowadays to enable users to discover exactly the information they need in the vast amount of data and to make it possible to integrate their large amount of image material efficiently. However, traditional content-based image retrieval techniques are based on images, and there is a “semantic gap” between this and people's understanding of images. To address this “semantic gap,” a keyframe image processing method for 3D point clouds is proposed, and based on this, a U-Net-based binary data stream semantic segmentation network is established for keyframe image processing of 3D point clouds in combination with deep learning techniques.

## Introduction

In recent years, computer vision technology, deep learning technology, and related intelligent equipment have been developed rapidly, and many scholars and researchers have conducted in-depth research on them. Among them, the semantic understanding of scene images can well combine the above three aspects and can become a current hot spot, especially the easier access to deep data has made the related research more active. Solving the problem of semantic understanding of scenes essentially means using the location and feature relationship of pixel points to segment, cluster, and recognize pixels, using mathematical theories and methods to process pixel data, adding human language expressions and descriptions to pixel data, and thus realizing the intelligence of machines and the understanding of reality (Li, 2022a).

Traditional intelligent devices such as unmanned vehicles and robots usually rely on two-dimensional (2D) vision with one or two eyes to accomplish semantic recognition of scenes, and current methods have achieved good results; however, there are still some problems with target semantic recognition based on 2D color images: the correctness of image recognition depends mainly on the quality of the acquired images, such as changes in camera parameters, ambient lighting changes (especially at night) can greatly affect the final recognition, and three-dimensional (3D) point clouds can solve this problem well because of the depth information collected (Li et al., 2021a); therefore, the combination of 2D and 3D is receiving more attention.

Vision is the most important and fundamental human behavior, with over 75% of external information relying on vision. If robots could be made to have the same vision as humans, some complex and high-risk tasks would be used to assist or replace them. From a bionic perspective, the use of cameras instead of the human eye for visualization is both the most scientific and practical means of doing so and the most effective (Li et al., 2021b).

The integration of optoelectronics, computer image processing, image processing, signal processing, and other science and technologies into the modern measurement technology is the product of twentieth-century technology development (Li, 2022b). It is in the industrial automatic detection, product quality control, reverse design, biomedicine, virtual reality, cultural relics restoration, human body measurement, and many other aspects have a wide range of applications.

The United States, Germany, Japan, and other advanced countries in the 3D measurement technology started earlier, since the end of the last century has a large number of theories and methods (Chen et al., 2008). In recent years, in order to adapt to the trend of digital, fast-response production, the life of the product is becoming shorter, the 3D model constructed and applied in many fields is becoming more fine and complex, and people's requirements for the product are becoming higher; therefore, in terms of measurement accuracy, stability, and industrial design, more attention is being paid to advanced foreign technology. In addition, the current 3D measurement technology has many shortcomings, especially on complex curved surfaces, where the material needs to be measured, and the level of adaptiveness and automation of the object's form factor is not high, so there are still many problems to be solved in practical applications. Therefore, this article focuses on the semantic understanding of keyframe images in 3D point cloud scenes, using deep learning techniques to combine 2D information with 3D depth information to investigate the semantic understanding of keyframe images in 3D point cloud scenes.

## Introduction to related technologies

### Deep learning

Deep learning is a comprehensive approach to model analysis that includes three different methods.

1. A network based on convolutional operations, called CNN (Cycle Network).
2. A self-coding neural network based on multilevel neurons, which incorporates two new coding methods, namely, autoencoder and sparse coding.
3. A multilevel self-coding neural network is used to pretrain the neural network, and the recognition information is combined with a deep trusted network to further optimize the neural network weights (He et al., 2002; Wang et al., 2002; Qie, 2010; Li et al., 2011).

After multilevel processing, the “low-level” feature representation is gradually transformed into a “high-level” feature representation, and then the “simple model” is used to perform complex classification and other learning tasks. Thus, deep learning can be understood as “feature learning” or “representation learning” (Peng et al., 2004).

In the past, the application of machine learning to practical work often required a specialist to characterize a sample, which led to “feature engineering.” It is well known that the quality of features is directly related to the performance of generalization, and it is not easy for human professionals to design good features; feature learning (feature learning) uses machine learning techniques themselves to generate good features, allowing machine learning to move further toward “unmanned data analysis” (Wan and Morshed).

In recent years, researchers have been gradually integrating these approaches, for example, by combining supervised learning-based convolutional neural networks with self-coding neural networks to form convolutional deep convolutional inverse (CI) networks by adjusting the parameters of the network with discriminative information without supervision. The deep learning approach is difficult to train due to the large number of model parameters required, while the general rule of statistical learning shows that as the model parameters increase, the training data required also increase (Lou, 2004).

In the 1980 and 1990s, the constraints of computer computing power and the level of technology that allowed for large amounts of data analysis prevented deep learning from being effective for pattern analysis. Restricted Boltzmann machines (RBM) became a powerful tool for enhancing the depth of neural networks after Hinton et al. proposed a fast CD-K-based method for computing the weights and biases of Boltzmann machine (RBM) networks, which led to the later mass application of deep belief networks (DBNs) (as developed by Hinton et al. and already used in speech recognition by companies such as Microsoft). At the same time, techniques such as sparse coding allow for the automatic extraction of data in deep learning. In recent years, convolutional neural networks based on local data have also been widely used (Zhang et al., 2017).

### Point cloud processing methods

The 3D digital image information obtained through 3D measurement devices is dominated by spatially discrete 3D point coordinate information. To obtain complete and accurate point cloud data, a large amount of 3D data needs to be processed before later surface reconstruction modeling is carried out (Song et al., 2017).

Point cloud processing includes topological reconstruction, point cloud denoising, point cloud smoothing, point cloud sampling, point cloud sorting, point cloud merging, point cloud encryption, and triangular meshing (Litany et al., 2017).

#### Point cloud noise reduction

Due to the influence of measurement equipment and other factors, there is inevitably some noise in the measurement data, resulting in a “rough burr” in the 3D display of the point cloud data. Noise has a significant impact on the modeling of the model.

If no noise is removed, the final object constructed will differ significantly from the real object due to the presence of noise points. Therefore, the first thing to do before processing the point cloud data is to denoise it (Lin, 2014).

#### Optimisation of point clouds

A simplified algorithm for point clouds is outlined in the paper by M. Pauly et al. (Feng, 2015). They are the basis for most current simplification algorithms. In this article, the simplification methods for point clouds are divided into three categories, namely, clustering, iteration, and granular simulation.

##### Aggregation

**Figure 1:**
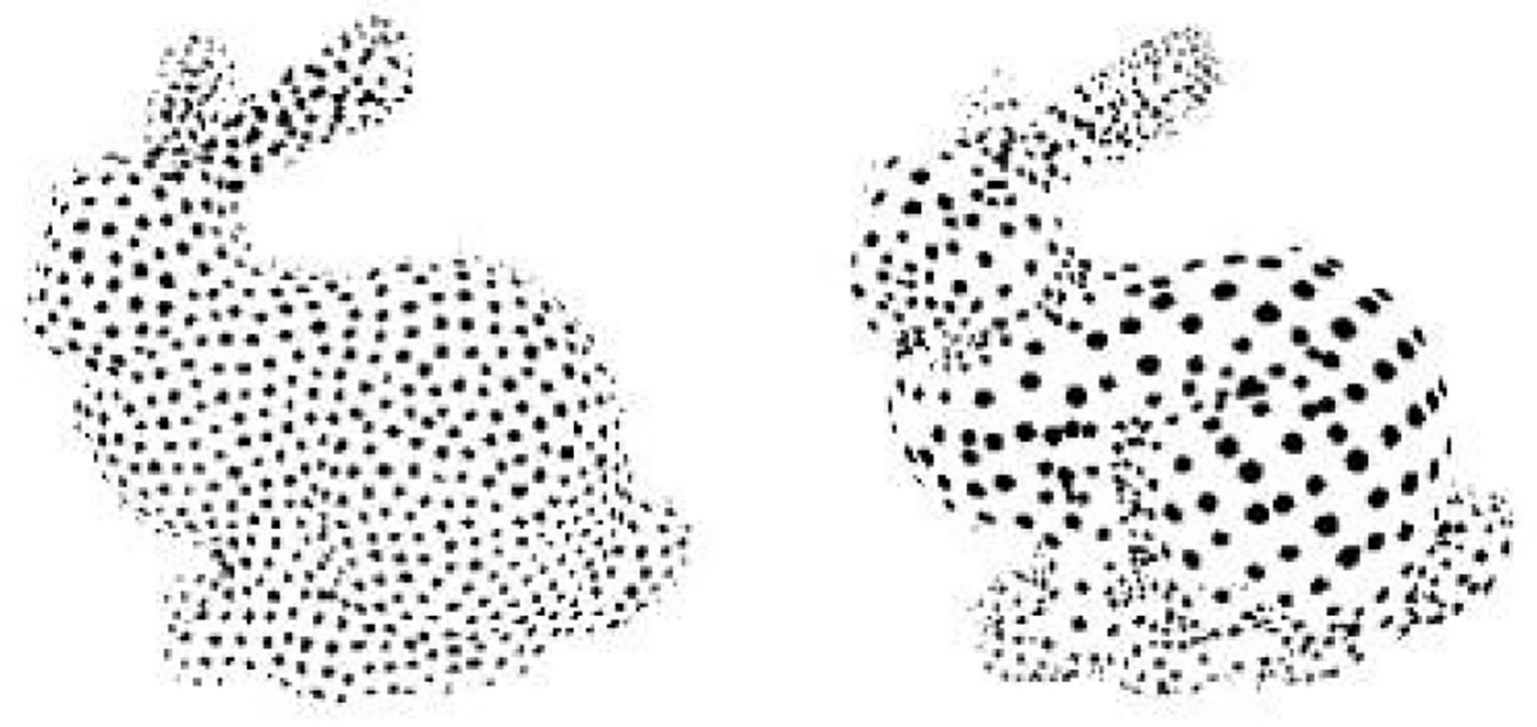
Clustering method instance.

Clustering is a common method for computing complex objects in three dimensions. The basic idea of clustering is partitioning. There are two main types, namely, bulk (envelope box) and planar partitioning. Bulk partitioning does not take the sampling density of the point cloud into account but allows for a good fusion of multiple envelopes, whereas the opposite is true for planar partitioning. An example of the clustering method is shown in Figure 1.

##### Iterative method

**Figure 2:**
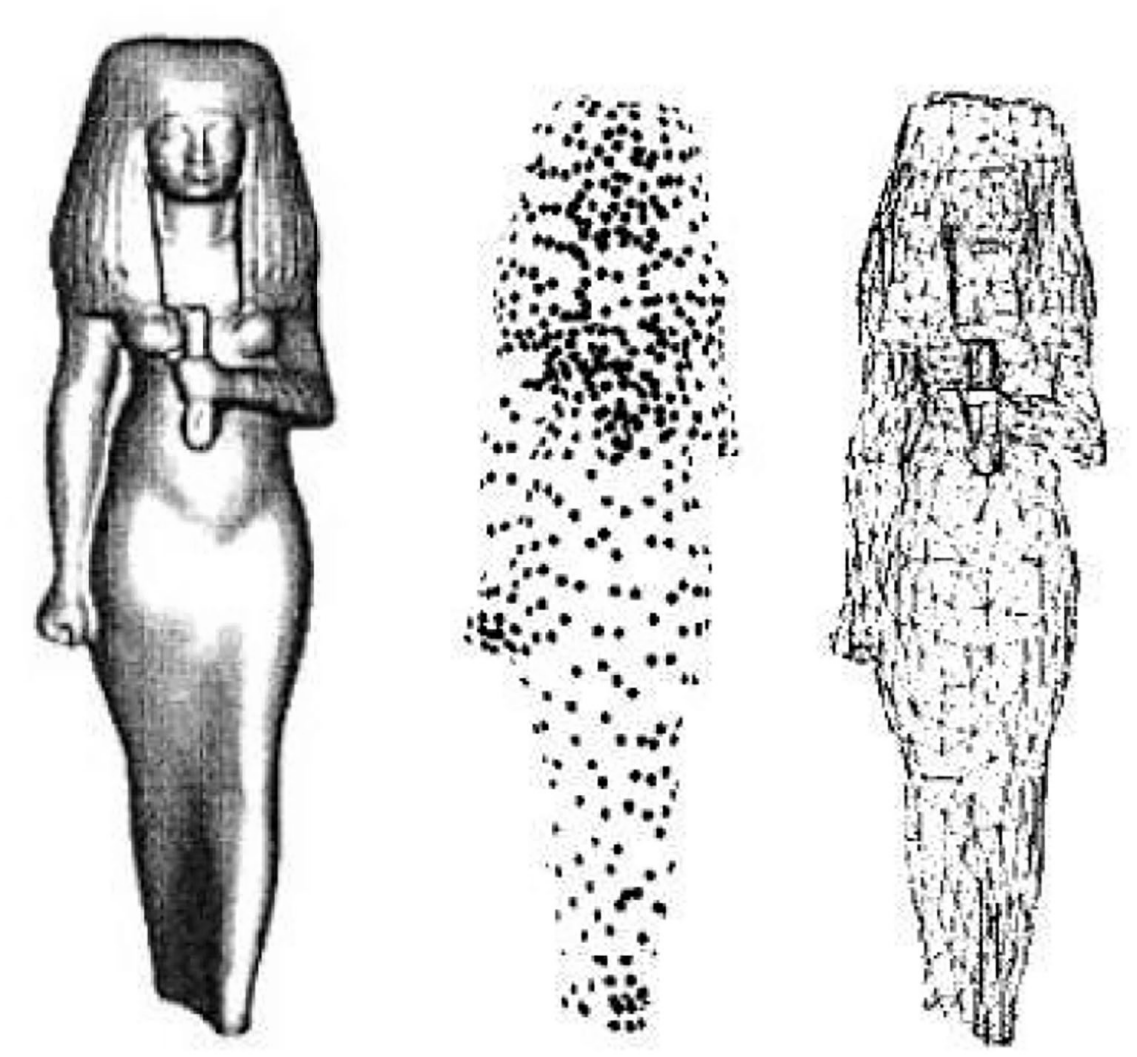
Iterative method instance.

As shown in Figure 2, this iteration is very similar to the simplification of asymptotic meshes. In simplifying the mesh, the vertices of each mesh were first assigned an error and then moved from small to large. The vertices are simplified, then each vertex is recalculated, and the vertices are removed to a tolerable range (Silberman and Fergus, 2011). This point iteration method makes the sampled point cloud only a subset of the original point cloud, and the results are not ideal. The error of a point is derived as a real symmetric matrix Q from one of the endpoints at which the point and its neighboring edges are located, such that the error of a point pair (edge) can be expressed as Qv = Qv1 + Qv2.

##### Particle simulation

**Figure 3:**
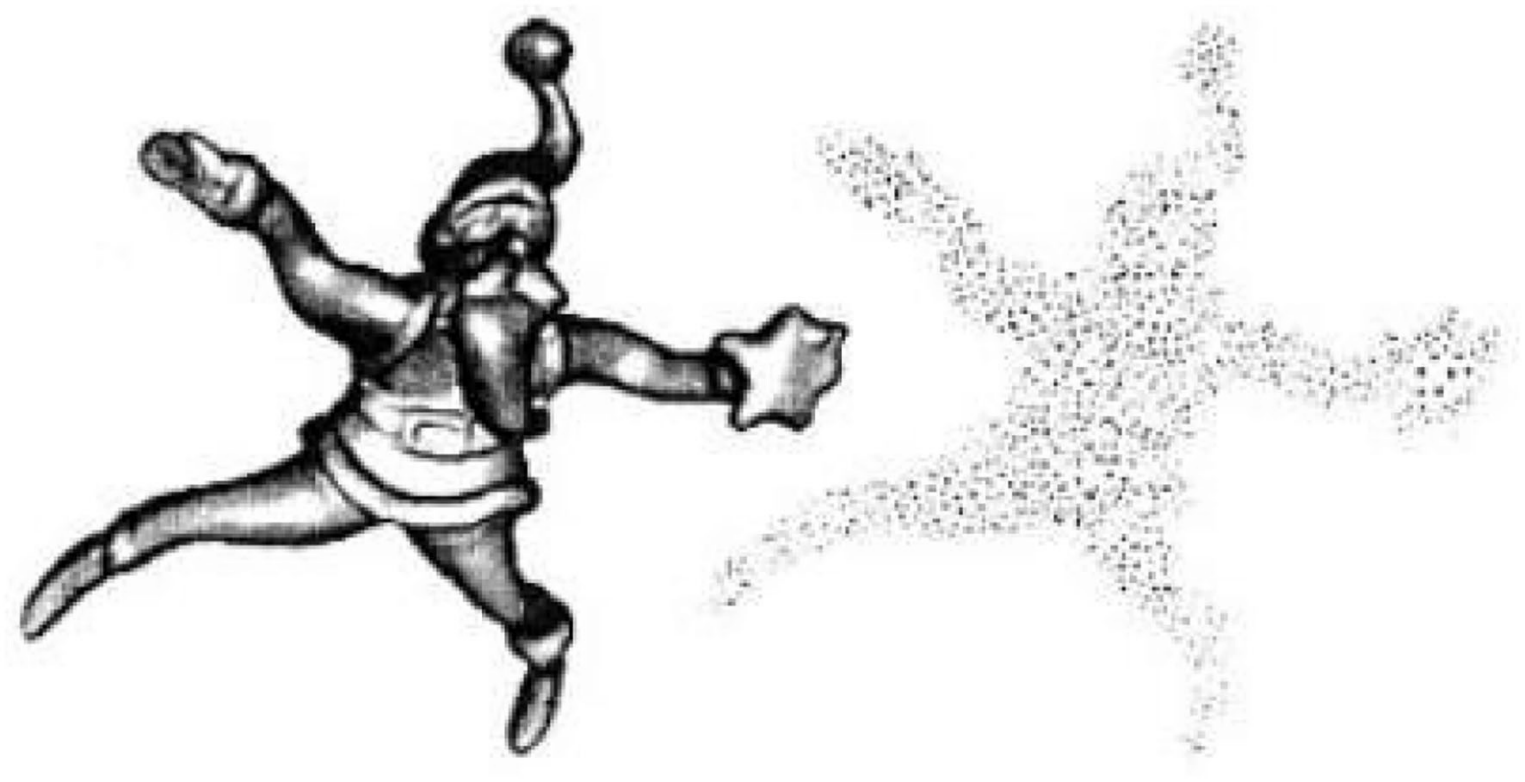
An example of simulation of particle.

Particle simulation is a method of sampling polygonal surfaces. Since the sampling points are taken on polygonal planes, the accuracy of the sampling is related to the area of the polygonal surface. Turk also uses a curvature estimation method, which allows the sample density to vary with curvature. The sampling of polygons is achieved by triangulating the sampling points. It is clear that the above point cloud resampling method is also feasible. The point cloud data can be replaced with points and their neighbors, and the area of the polygon surface corresponds to the neighboring area of the point cloud. An example of its particle replica is shown in Figure 3.

From the above three methods for comparison, because the method proposed in this article is based on deep learning to conduct research, clustering and particle simulation are not too suitable for training performance improvement, so this article chooses the iterative method to simplify the point cloud.

## Application method design

### Method of generating semantic point clouds for keyframes

**Figure 4:**
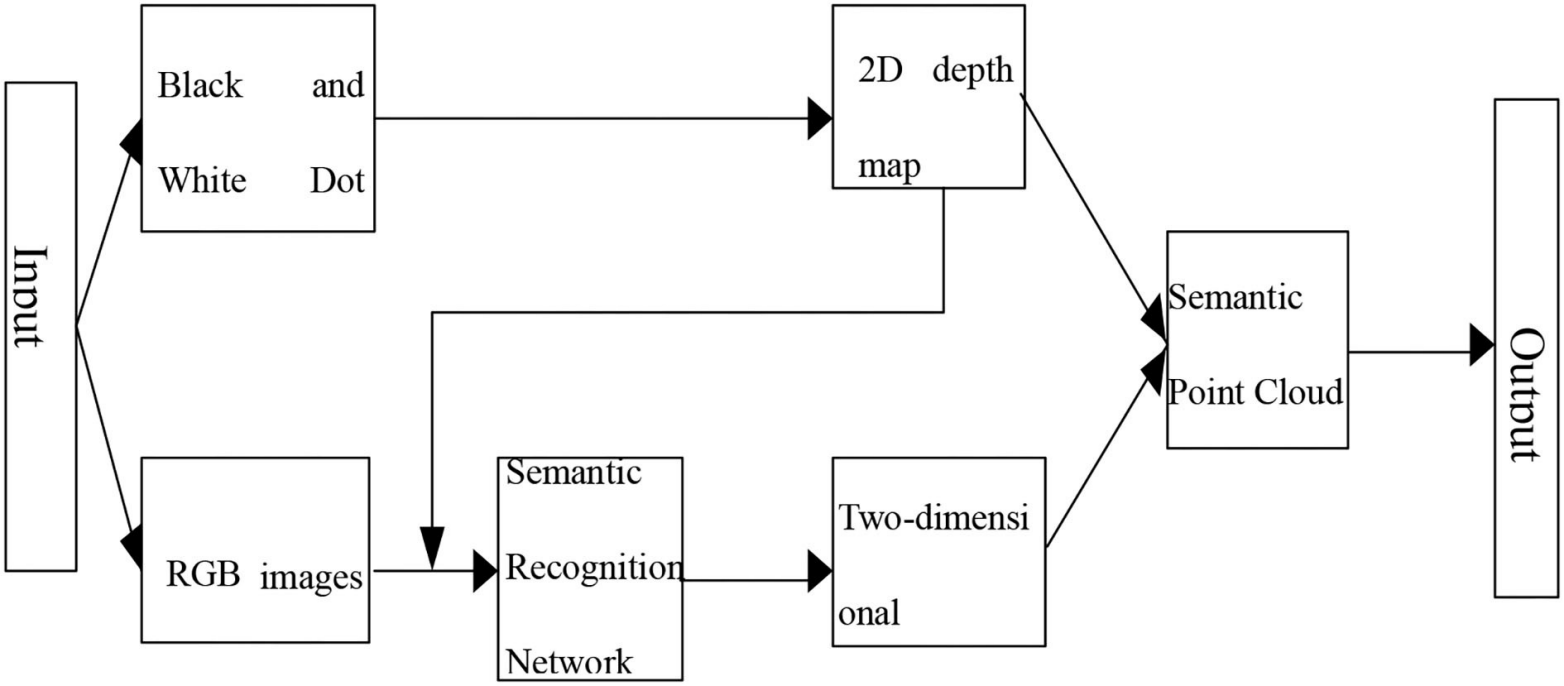
Flowchart for generating a semantic point cloud of keyframes.

This part obtains point cloud semantic data by semantically dividing the point cloud data through the coordinate transformation relationship between the point cloud data and the depth map. The flowchart for generating keyframe semantic point clouds is shown in Figure 4.

Several data types commonly used in point cloud data are XYZ and XYZRGB. To make the semantic information better presented, we selected the XYZRGB data type, divided the different semantic categories into *RGB species* standards, and visualized the semantic classes by their corresponding semantic information to RGB channels.

As shown in Figure 4, in order to generate semantic point clouds, first, *Point x*(_*w*_, *y*_*w*_, *z*_*w*_) in the point cloud data is transformed into each point *Depth* (*u, v*), and finally, the 3D data are transformed into 2D image depth; on this basis, each pixel *P* (*u, v*) of the depth map is transformed into semantic point cloud data points, semantic point cloud data points *Point* (*x*_*w*_, *y*_*w*_,, *z*_*w*_, *r*_*w*_*, g*_*w*_*, b*_*w*_), and finally the key frame semantic point cloud (Hinton, 2006).

### Dual data stream semantic segmentation network design

To make full use of RGB and depth information, the impact of the image on the process of semantic understanding by factors such as changes in camera parameters and uncontrollable indoor lighting is reduced, and the problems of image data such as occlusion, incomplete data, disorder, difficulty in feature extraction, a large amount of data, large changes in scene type, and unclear background are reduced, thus reducing the impact on semantic understanding (Yu et al., 2013).

**Figure 5:**
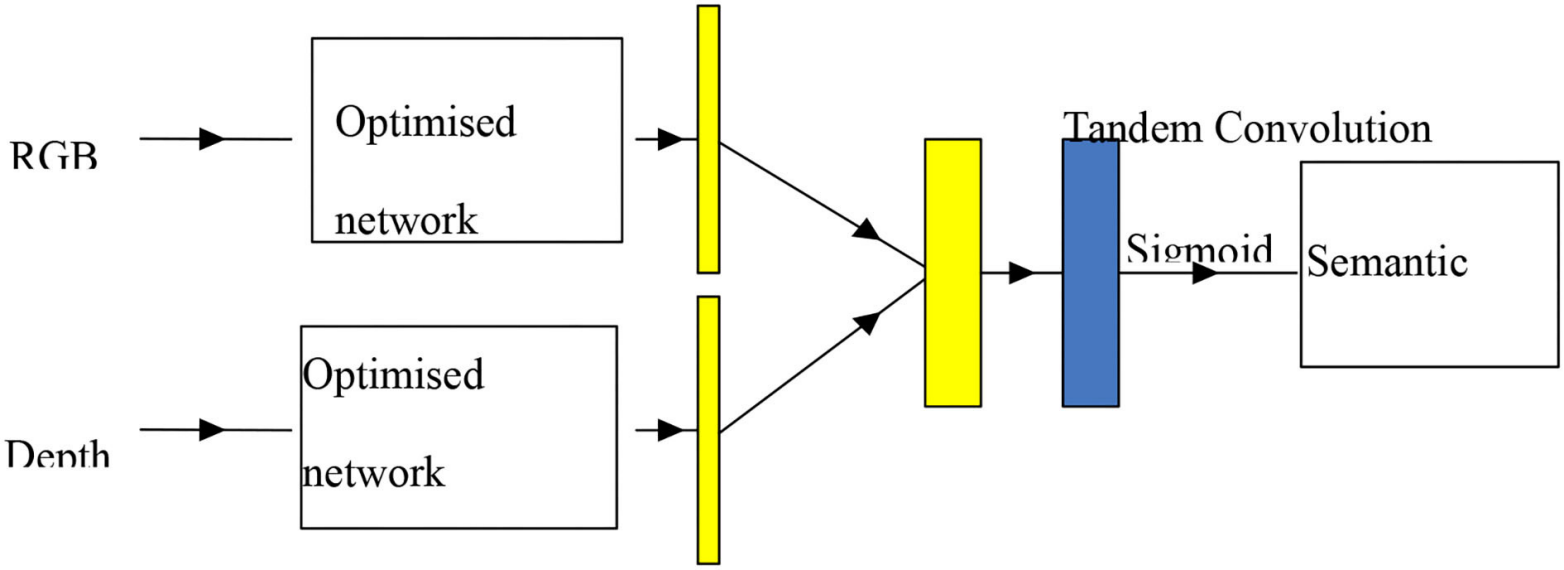
U-Net-based semantic segmentation network for dual data streams.

The diagram of the U-Net-based dual data stream semantic segmentation network model is shown in Figure 5, which is divided into three parts, namely, RGB data stream training channel, depth data stream training channel, and contribution decision layer. When RGB and depth data stream training are two separate systems that do not intersect with each other, the system uses the optimal U-Net network structure as described in the previous section to ensure the effectiveness of individual feature learning while preventing false interference with each other. Based on this, the abstract segmentation properties of both RGB and depth images are combined to form a weighted array of thresholds that measure the role of RGB and depth at individual pixel points, thus enabling semantic segmentation of the target (Lu and Zhang, 2016).

**Figure 6:**
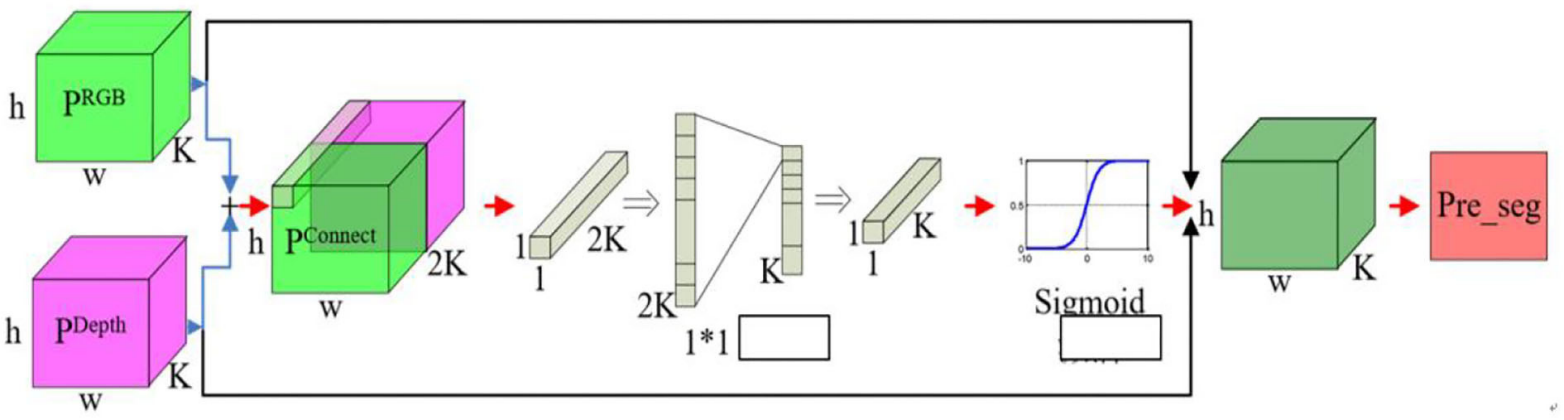
Contribution trade-off prediction process diagram.

The optimal U-Net network structure was used in order to make output probability profiles for RGB and depth data streams with *K* representing the classification number, *h* representing the height of the segmented image, and *w* representing the width of the segmented image. The contribution rate trade-off predictions are shown in Figure 6.

The use of U-Net technology achieves a direct combination of RGB and depth data, eliminating the original errors that exist between RGB and depth data, eliminating the interaction between RGB and depth information, and achieving a complementary advantage of the two.

The U-Net is a variation of a fully convolutional neural network similar to the one whose structure was drawn by the authors as a U. The network consists of two main classes, namely, search paths and extension paths. The search path is mainly used to obtain the background information in the image, while the symmetric extension path is used to precisely determine the regions in the image.

The U-Net is improved from fully convolutional networks (FCN), which does not encode and code the image as FCN does. For accurate localization, U-Net combines image features extracted from compressed paths with a new feature map (in more general terms, it fuses local and overall information to improve the prediction accuracy of object prime point classification). To allow the network to work more efficiently, it does not have a full connectivity layer, which allows for a significant reduction in the parameters required for training and also allows for the preservation of all information in the image through a unique U-shaped structure.

In the compression path, every two 3 × 3 convolutional layers (the largest 2 layers) are combined with the largest 2 × 2 sinks (2), and a relu start function is used after each layer to reduce the sampling of the original image, in addition to adding a cup of channels for each down-sampling.

For up-sampling, each step has 2 × 2 convolutional layers (the activation function is also relu) and two 3 × 3 convolutional layers, while a feature map (cut to the same shape) is added from the relative compressed path for each up-sampling step.

The method converts the feature vector of 64 channels into the desired number (e.g., 2) and, finally, the whole network of U-Net has 23 convolutional layers. The best feature of U-Net is its ability to convolve images of any size, especially for images of any size.

### Image processing

#### Removing image noise

**Figure 7:**
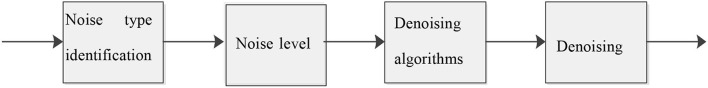
The flowchart of image denoising.

Before noisy images can be processed, two issues must be clarified, namely, the type of noise interference suffered and the degree of noise interference suffered. This method not only overcomes the blindness of traditional methods but also provides some guidance for the research on adaptive image noise reduction methods. In Figure 7, the correct noise removal process is shown.

Filtering of image noise requires the filters used to remove the noise while maintaining as much detail as possible. Image noise can be divided into two types. The first type is electronic noise, a noise caused by the random thermal movement of electrons in resistive elements, which is usually modeled using zero-mean Gaussian white noise, a method with a histogram of Gaussian functions and a flat power spectrum. The second type is particulate noise (pepper noise) due to improper exposure of the photographic plate.

The image is the first wavelet decomposed using wavelets to obtain the high-frequency coefficients HH of the noisy image, and its energy concentration is analyzed to finally determine if the image has Gaussian noise or pepper noise.

Noise removal has the following steps.

1. Compare the starting part of the computed wavelet with the initial data.
2. Obtain a factor of C. This factor represents the similarity of this type of information to the wavelet and increases as the correlation coefficient increases.
3. Move the wavelet to the next bit and repeat step 1 and step 2 until all the original data are completely covered.
4. Release the wavelets and repeat steps 1, 2, and 3.
5. Threshold filter all the resulting subwave coefficients.
6. Obtain the image information after noise using subwave synthesis of the filtered coefficients.

#### Remove the background of the image

When acquiring a target image, it is inevitable that information other than the target will be captured, the so-called background information. Not only is the background information of no practical value when acquiring point cloud 3D data, but it also generates noise, which affects the synthesis of the point cloud. It is, therefore, necessary to remove the background before point cloud synthesis can be performed, and the key to removing the background is to find the contours of the target itself. The contours are searched based on edge extraction techniques.

### Image edge check detection and extraction

Edge extraction is a fundamental technique in current image analysis and is the first step in the analysis and understanding of an image.

The method uses information about the extremes of the first-order differentiation of the image and the per-zero points of the quadratic differentiation to perform edge extraction. Specifically, when the change in the image is slow, the gray scale of its neighboring pixels does not change greatly and, therefore, its gradient magnitude is small (close to zero), while at the edges of the image, the location of the edges can be obtained using the magnitude of the primary differentiation magnitude as the gray scale of the neighboring pixels changes greatly. Similarly, the sign of the second-order derivative can be used to determine whether the edge of a pixel is the bright side or the dark side, while the zero point is the edge.

The first-order differential boundary operators include the Robert operator, Sobel operator, Prewitt operator, and Creech operator, while the Laplace operator and Gauss-Laplace operator are the second-order differential boundary operators.

In this article, the Canny boundary detection method based on the Laplace function is used.


$$\begin{array}{l} Laplace\left(f\right)\equiv \frac{\partial^{2}f}{\partial f^{2}}+\frac{\partial^{2}f}{\partial y^{2}} \end{array}$$

The first derivatives of the above equations are along the x and y directions. They are then combined into four directions of differentiation. The local extremum of the differentiation in these directions is a candidate for the boundary.

**Figure 8:**
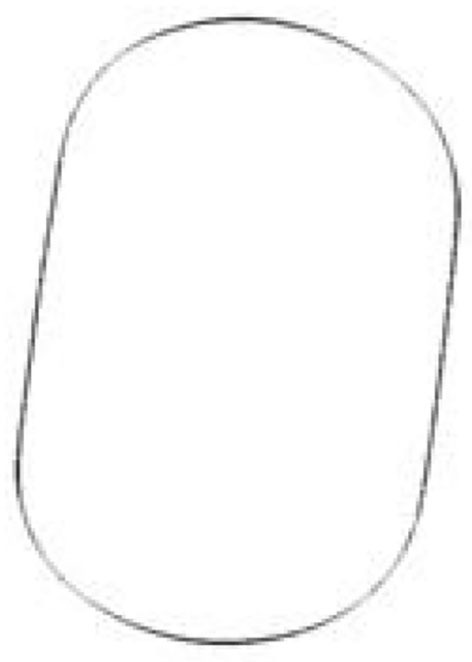
Outline of the figure.

After the boundary has been obtained, it needs to be segmented, where two thresholds are set, namely, upper and lower. If the gradient of a pixel exceeds the upper threshold, it is considered an edge, and images below this threshold cannot be recognized. Between these two boundaries, if it is connected to a point that exceeds the threshold, then it can be considered an edge. Canny suggests that the threshold ratio of upper and lower thresholds should be 2:1 to 3:1. The extracted profiles are shown in Figure 8.

After acquiring the profile of the target, some unsatisfactory situations may arise, and it is then necessary to fine-tune the resulting profile. Here, we are going to use Sneck's energy profile.

The energy in Sneck's model enables the curve to be smooth and continuous, while the external energy makes its positioning more precise. It is modeled as follows:


$$\begin{array}{l} E_{contour}=\alpha *\;E_{elastic}+\beta *\;E_{bending}+\gamma *\;E_{external} \end{array}$$

The energy equation shows that E_contour_ contains three components, namely, the elastic energy *E*_*elastic*_, the bending energy *E*_*bending*_, and the external energy *E*_*elastic*_. The elastic energy and the bending energy are called the internal energy.

Generally, α, β, γ is adjusted to control the change in the Snake model. In contrast, the elastic energy E_elastic_ in the above equation is generally expressed as the Euclidean distance between two points, and the bending energy E_bending_ is expressed as


$$\begin{array}{l} E_{bending}={\left|P_{i+1}-2P_{i}-{P_{1}}_{-i}\right|}^{2} \end{array}$$

where P_i+1_,p_i_, and p_i−1_ are the three neighboring points. The final external energy is expressed in this article as the distance from the point to the geometric center, i.e.


$$\begin{array}{l} E_{external}=\sqrt{{\left(P_{ix}-M_{x}\right)}^{2}+{\left(P_{iy}-M_{y}\right)}^{2}} \end{array}$$

where *P*_*ix*_ and *P*_*iy*_ are the current points and M_x_ and M_y_ are the geometric centers of the contour lines.

**Figure 9:**
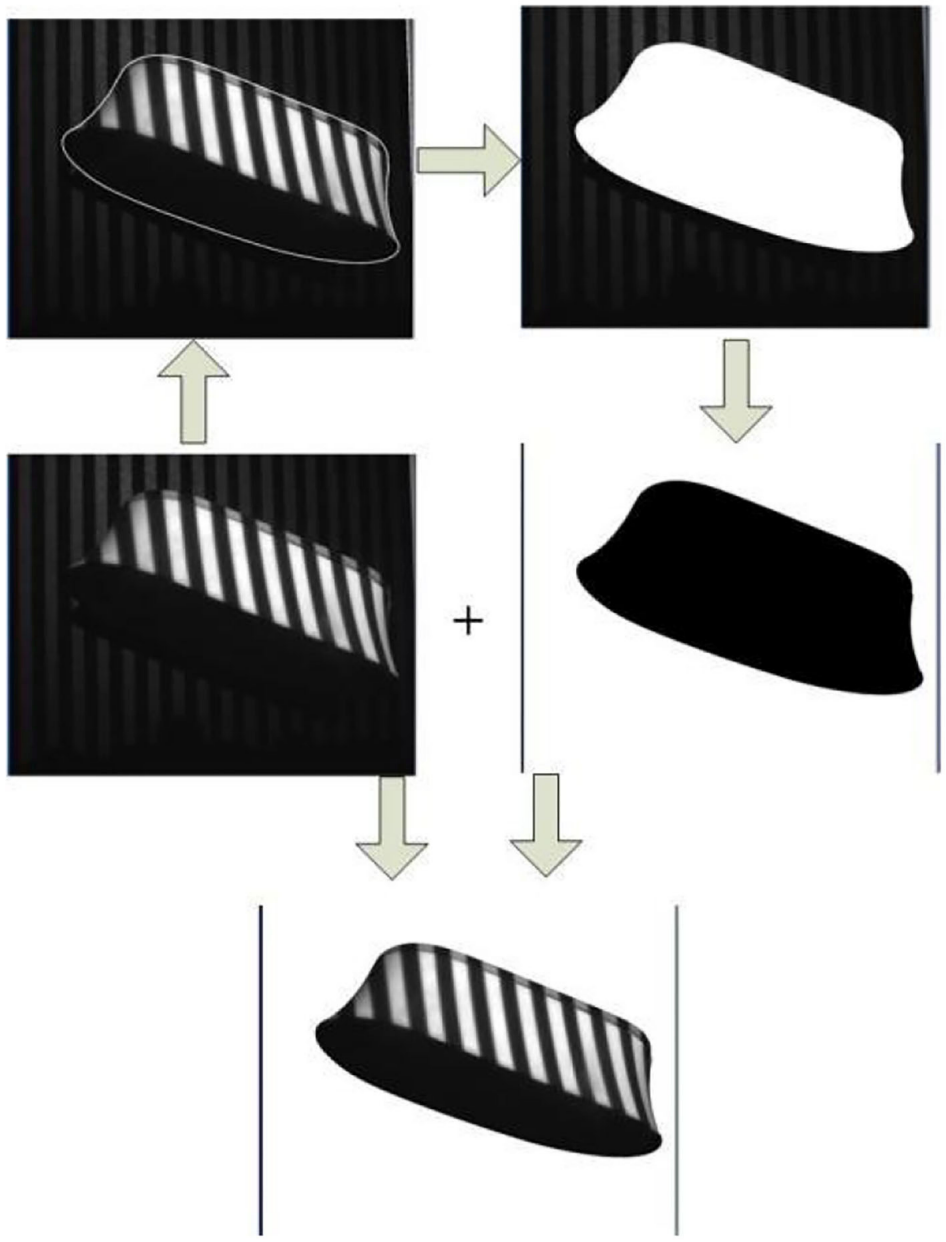
Remove the background.

The shape of the object can be well adjusted by adjusting the parameters of the Sneck curve mentioned above. After the contour lines have been obtained, they are binarised and summed with the original image to obtain an image containing only the object itself with a white background. The process is illustrated in Figure 9.

## Experimental analysis of application practice

### Description of the experimental process

For the problem of semantic segmentation in 3D scenes, this part focuses on the description and processing of experimental data and the evaluation and analysis of practical results to test the proposed method in this article.

Finally, the generation of key frame-sense point clouds and the 3P-ICP algorithm were verified using the Microsoft kinect_V1 acquisition device, the 3D semantic scene was reconstructed, and the experimental results were summarized and analyzed, proving that the methods and algorithms described in this article are feasible and effective.

### Description of the experimental dataset

Regarding RGB-D scene data, a number of datasets were published between 2012 and 2017, including NYUDv2, SUNRGB-D, SceneNN, 2D-3D-S, SUNGG, SceneNetRGB-D, Matterport3D, and ScanNetData, and were analyzed and collated according to dataset type, whether they contained color images, depth images, 2D semantic labels, scene size, label category, scene type, and data size. The results of the publicly available dataset statistics are shown in Table 1.

**Table 1:** Statistical results of publicly available datasets.

**Name**	**NYUDv2**	**SUN RGB-D**	**SceneN N**	**2D-3D-S**	**SUN GG**	**SceneNet Matterport3D RGB-D**		**ScanNet Data**
**Authenticity**	**Real**	**Real**	**Real**	**Real**	**Synthesis**	**Synthesis**	**Real**	**Real**
Time	2012	2015	2016	2017	2016	2017	2017	2017
RGB	Yes	Yes	-	Yes	None	Yes	Yes	None
Depth	Yes	Yes	Yes	Yes	None	Yes	Yes	Yes
Semantic	Yes	Yes	Yes	Yes	None	Yes	–	Yes
Size	640*480	–	640*480	1,080*1,080	–	320*240	1,280*1,024	968*1,296
Number of categories	894	800	–	13	84	255	40	–
Number of types	26	47	–	11	24	5	3	1513
Number of photos	1449	10,335	–	70,496	–	5M	1,94,400	2.5M

### Semantic segmentation network model experiments

Before conducting the semantic segmentation network model experiments, its parameters are set as follows: amount of training data: 60,000; mini-batch method: batch_size = 100 number of iterations; iteration = 30,000; average number of iterations per epoch: 60,000/100 = 600. When the iteration proceeds to 600 times, it is considered to have completed an epoch. Through long-term training, the samples were predicted using the evaluation index, and the final conclusions were as follows: the proportion of pixels in the multiclass segmentation task varied widely, with an average distribution between 1.1 and 24.0%, but the accuracy and recovery rates for each classification were high, and the network model attenuated the effect of the proportion of pixel types on the segmentation effect. The algorithm achieved better results in terms of pixel accuracy, pixel prediction accuracy, and average predicted intersection ratio, objectively demonstrating the correctness of the method. The statistics of the final evaluation indexes are shown in Table 2.

**Table 2:** Statistics of the final evaluation index results.

**Class**	**% of pixel possession (%)**	**mean_presition (%)**	**mean_recall (%)**	**Acc (%)**	**ave_acc (%)**	**mean_IOU (%)**
0-others	23.9	72.5	72.5	73.4	68.6	56.7
1-ceiling	1.1	77.8	82.2			
2-wall	24.0	89.6	86.1			
3-floor	13.3	89.4	80.5			
4-window	6.6	75.3	51.8			
5-door	2.7	55.9	43.4			
6-table	11.2	72.3	62.7			
7-char	7.3	64.4	82.9			
8-sofa	2.1	57.6	31.9			
9-cabinet	5.0	54.7	43.9			
9-monitor	2.8	65.0	50.1			
Total-have0	100	76.6	71.5		–	
Total-no0	76.1	77.9	71.2			

**Figure 10:**
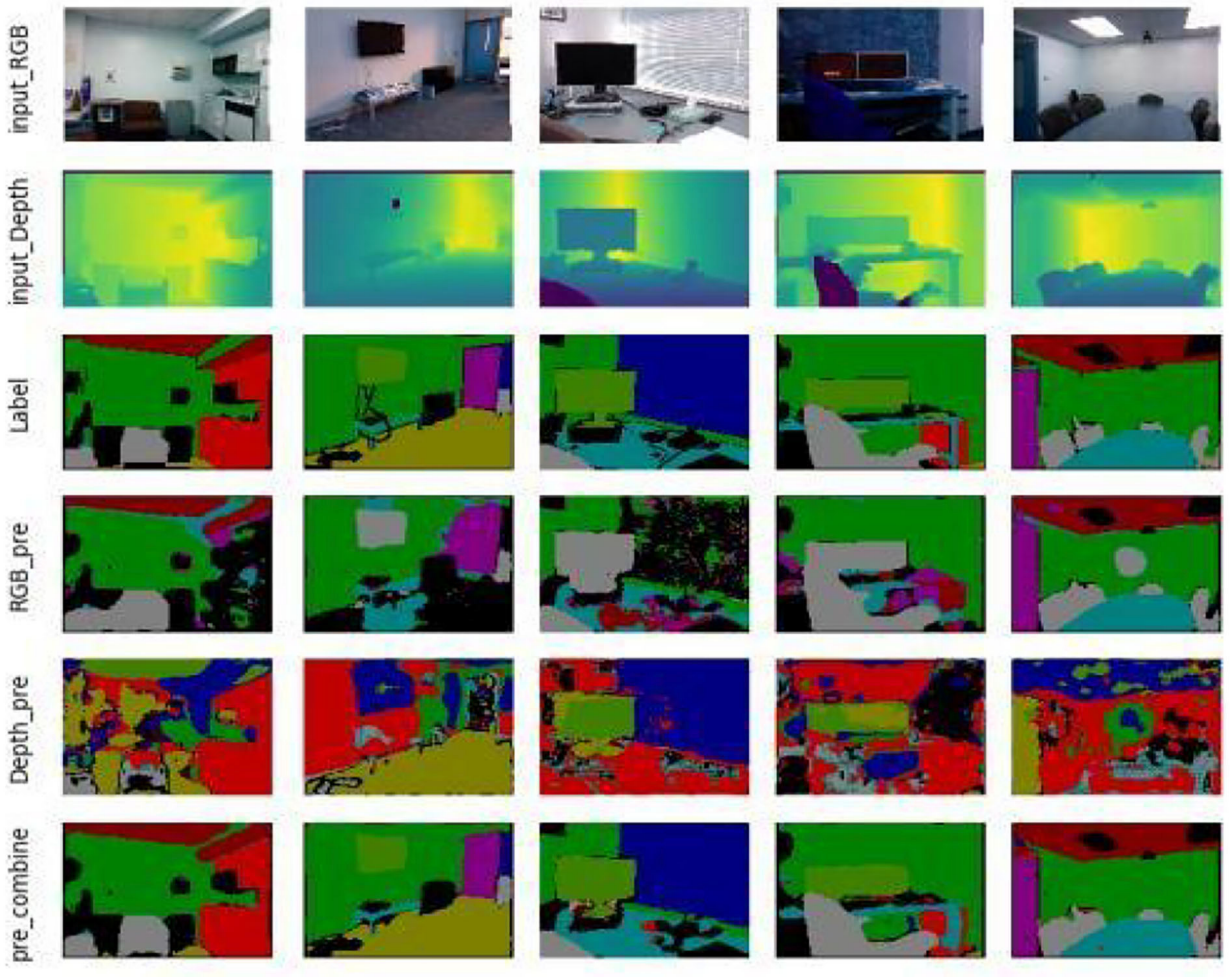
Semantic segmentation results for test data.

**Figure 11:**
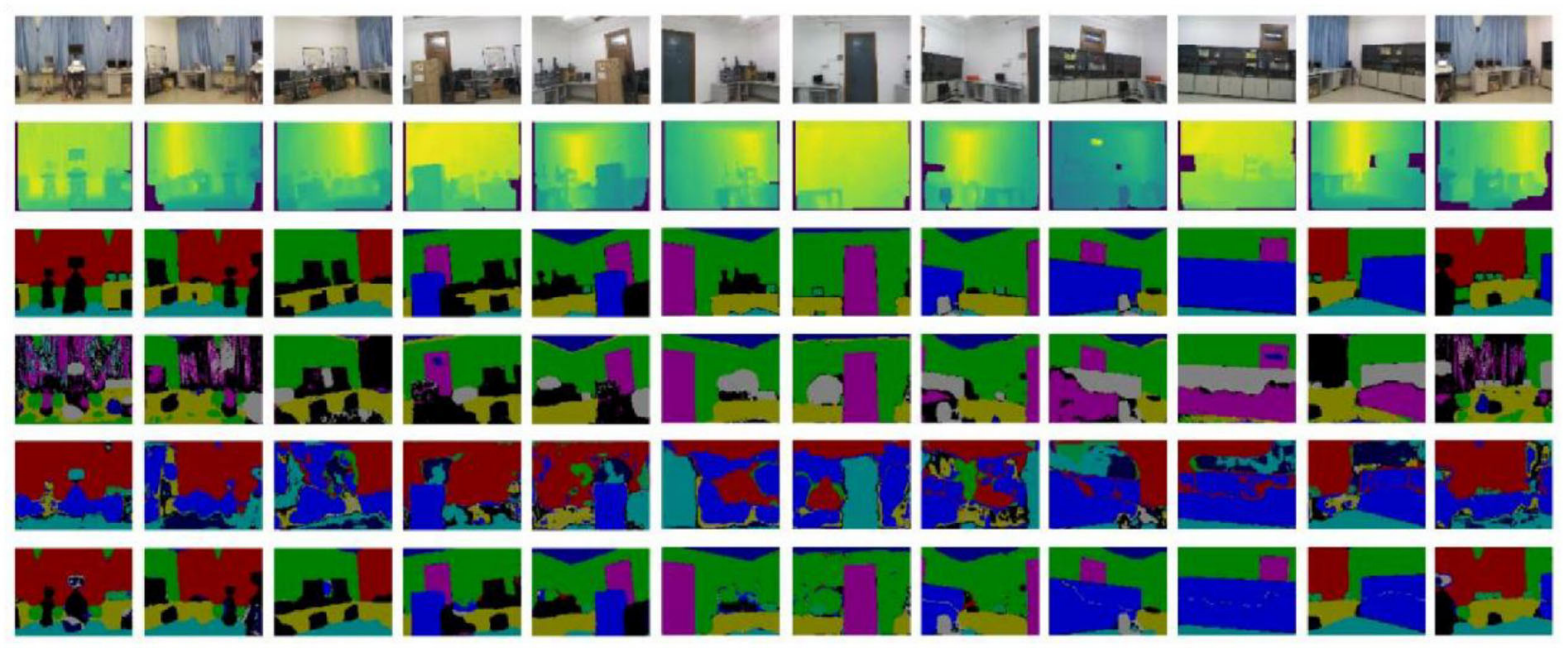
Semantic segmentation results of the actual collected data.

Based on this, the semantic partitioning of the two data streams is analyzed using the semantic partitioning network model built by U-Net, and the corresponding semantic partitioning results are given. In Figure 10, the first line is the input RGB image, the second is the depth image, the fourth is the prediction of the characteristics of the RGB data stream path, the fifth is the depth data stream channel feature prediction, and the last line is a network model based on the semantic division of the two data streams by U-Net. Visually, it can be seen that the network has good semantic segmentation capabilities, while the color RGB and depth data streams show significant differences in different classifications based on the data characteristics, for example, the depth information features of the wall type are not significant, so the learning of the whole network relies mainly on RGB color images. In the curtain type, features such as the depth waveform of the depth map data are more significantly represented than in the color images and, therefore, the final result depends on the depth map. By classifying two different data streams, RGB and depth, and fusing their features, better results were obtained. The semantic segmentation results for the test data and for the actual acquisition data are shown in Figures 10, 11, respectively.

**Figure 12:**
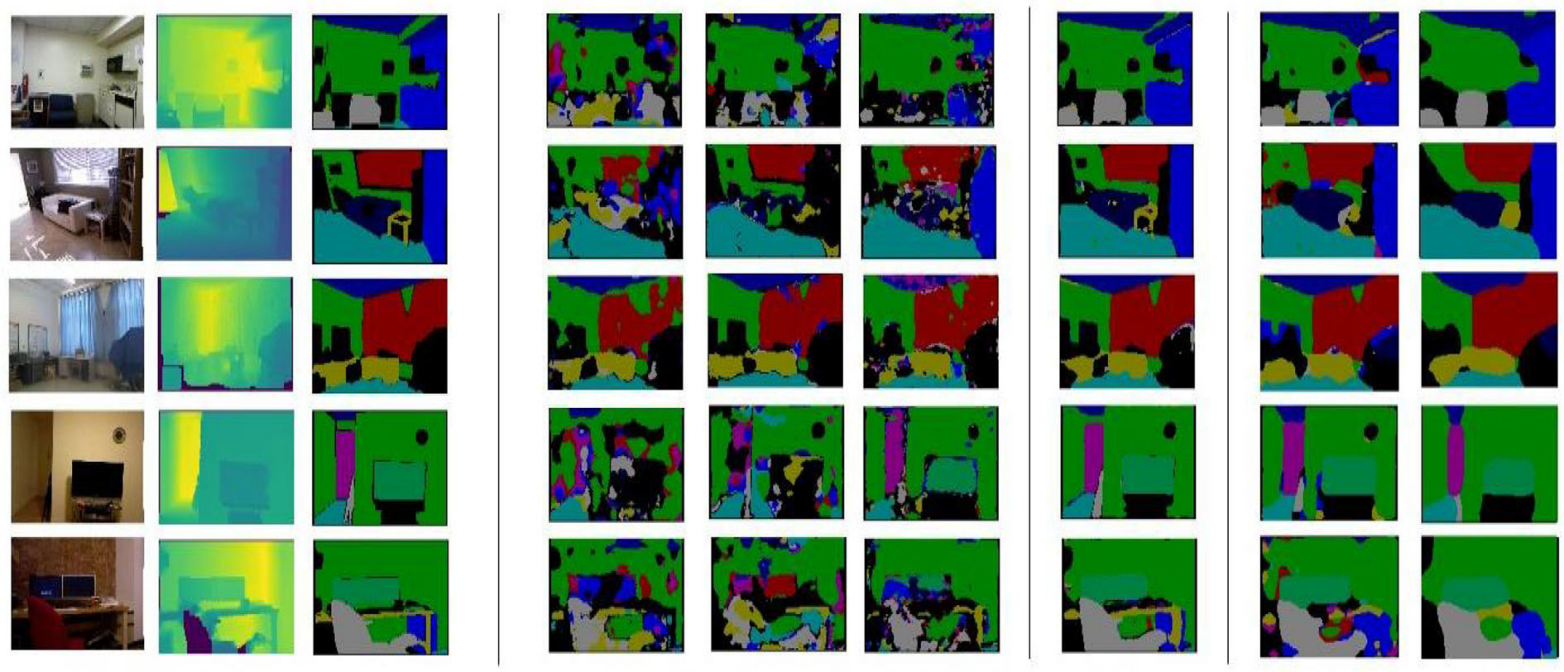
Comparison of segmentation results of dual data streams with different channels of data.

To further evaluate the effectiveness of the U-Net-based semantic segmentation network for dual data streams, this article compares three data types, namely, RGB three-channel data, depth single-channel data, and RGB-D four-pass data, using the U-Net technique, and comes up with the optimal solution for both data streams. A comparison of the segmentation results between the dual data streams and the different channel data is shown in Figure 12, and a comparison of the evaluation metrics between the dual data streams and the different channel data is shown in Table 3.

**Table 3:** Comparison of evaluation metrics for dual data streams and different channel data.

**Data Channel**	**Acc (%)**	**ave_acc (%)**	**ave_ mean_IOU (%)**	**mean_presition(No 0) (%)**	**ave_mean_recall(No 0) (%)**
Depth	53.8	49.5	37.5	60.8	53.4
RGB	59.7	54.7	43.3	65.7	57.1
RGB-D	65.8	59.6	46.9	70.1	64.2
Ours	73.4	68.6	56.7	77.9	71.2

Finally, a secondary data stream semantic segmentation was performed on the FCN, the original U-Net network using this training data, as shown in Figure 12 and Table 4. In Figure 12, the segmentation results of the original U-Net are shown with an equal scale enlargement of the size 640^*^480. The segmentation results of the FCN have good boundaries and poor edge quality, and the segmentation is low in all metrics; moreover, because of the difference in size and quality compared to the original U-Net compared to the optimized U-Net network, its output image size and quality were improved, while mean_IOU increased by 7%.

**Table 4:** Comparison of evaluation metrics for different networks using dual data stream training methods.

**Network**	**Acc (%)**	**ave_acc (%)**	**ave_ mean_IOU (%)**	**mean_presition (No 0) (%)**	**ave_mean_recall (No 0) (%)**
OID-U-Net	67.0	64.2	49.4	71.2	65.4
FCN	47.8	44.1	35.9	51.1	48.9
Ours	73.4	68.6	56.7	77.9	71.2

### Results and analysis of the keyframe semantic point cloud generation method

For the Microsoft kinect_V1 acquisition device, the device parameters were midpoint *u*_0_ = 325.5, *v*_0_= 253.5, focal length *f*_*x*_ = 518.0, and *f*_*y*_= 519.0.

**Figure 13:**
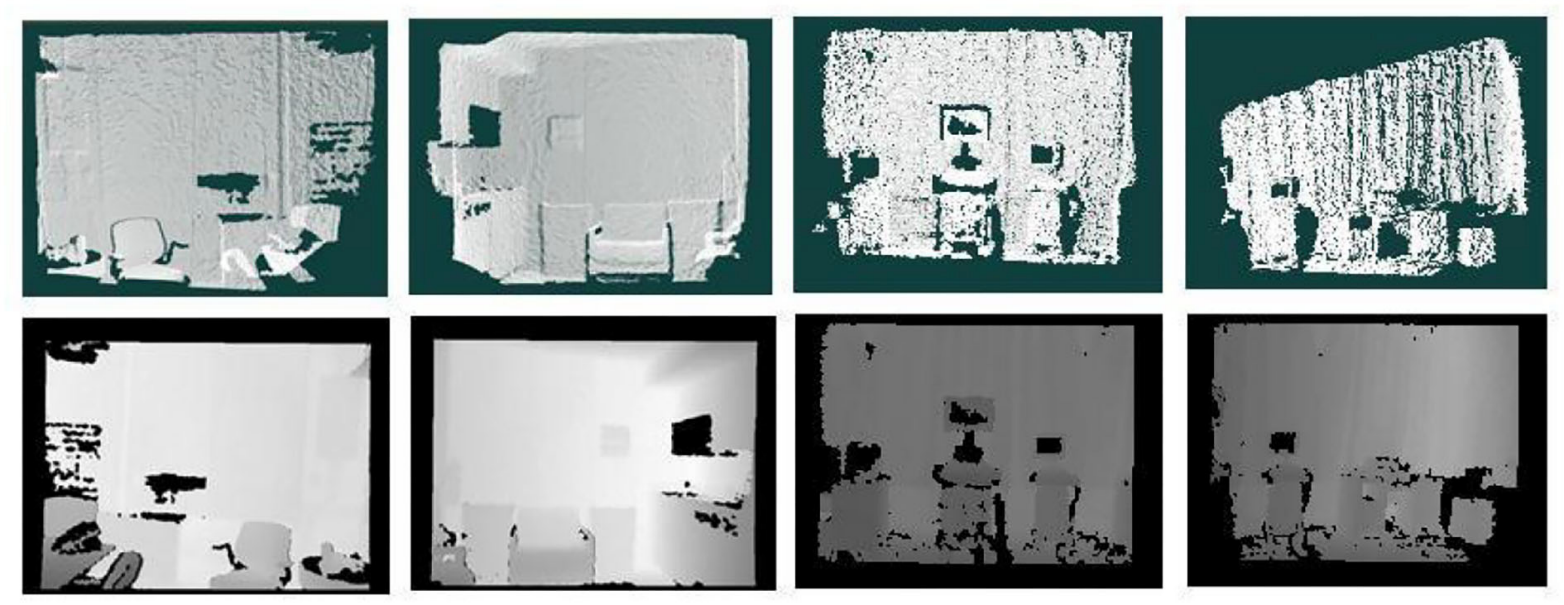
Conversion of 3D point cloud data to 2D depth map data.

According to the conversion of point clouds to depth maps, the black and white point clouds were first converted into depth maps, the effect of which is shown in Figure 13, which realizes the conversion of 3D point clouds into 2D depth maps.

**Figure 14:**
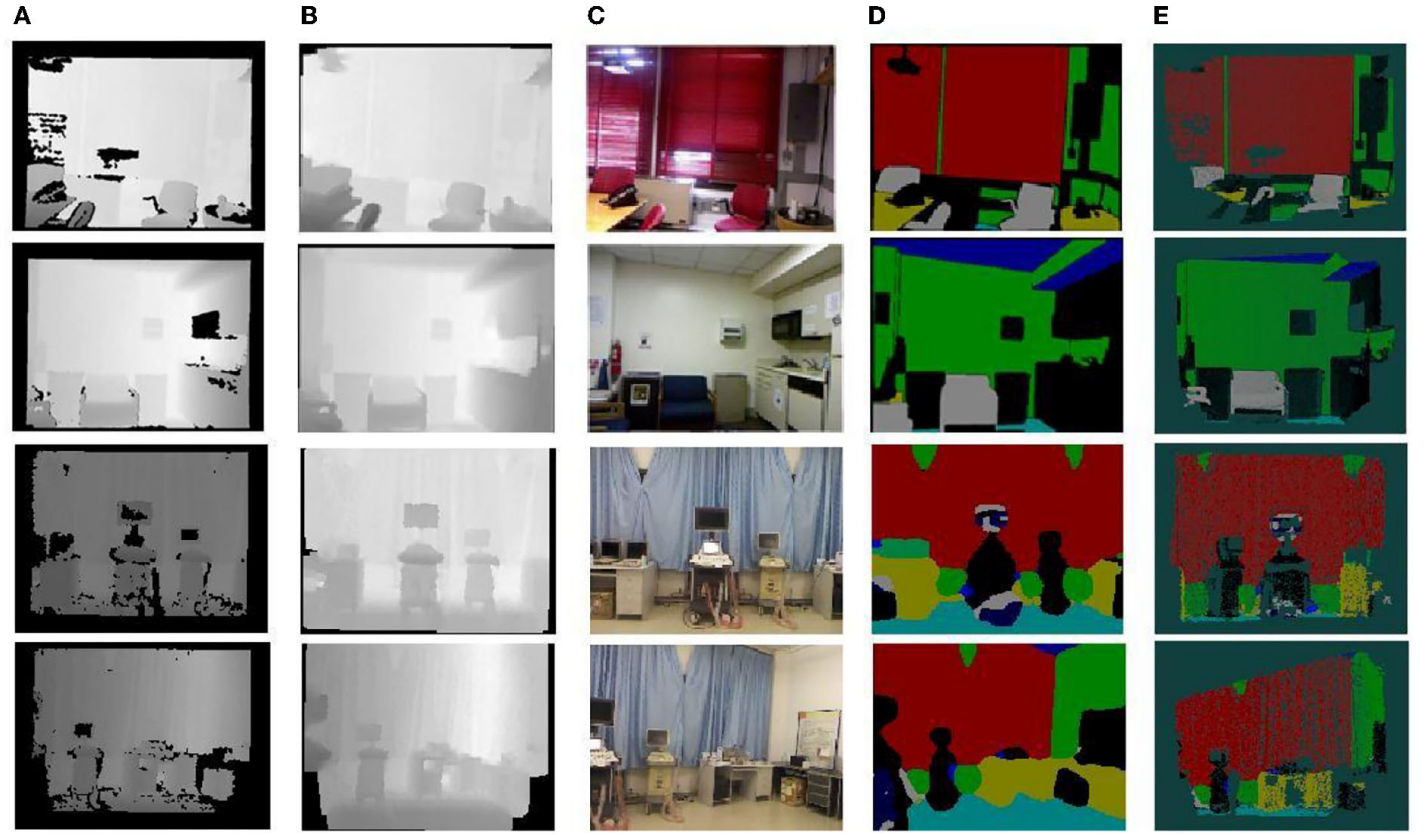
Example of semantic segmentation results. **(A)** Depth map. **(B)** Depth map filtering. **(C)** Color map. **(D)** 2D semantics. **(E)** Semantic point cloud.

An example of the results of semantic segmentation is shown in Figure 14. The diagram is divided into five sub diagrams a, b, c, d, e, which are the results of the segmentation in different periods, and the five sub diagrams together form the segmentation process. A multiscale bidirectional filtering of the images was performed and then combined with color photographs to form a U-Net-based semantic segmentation network to obtain 2D semantic segmentation results, which are displayed in different colors, where the first action detects the sample, the second line indicates the predicted result of the actual data, subfigure (a) is the depth map after the transformation of the point cloud data, subfigure (c) is the acquired color photographic data, and subplot (d) is the input of (b) chart data and (c) to a U-Net-based dual data stream semantic segmentation network model, resulting in a multicolour display. Finally, the formulation is used to semantically segment the image and transform it into a 3D point cloud with the corresponding categorical colors, as shown in subfigure (e), to obtain a keyframe semantic point cloud.

## Conclusion

The semantics of images are the key to understanding the real-world machine learning. In terms of in-depth data acquisition, this thesis focuses on the semantic understanding of 3D point clouds and gives a semantic-based 3D model based on this. In terms of semantic segmentation, the U-Net technique is used to achieve semantic segmentation of two data streams, and the relationship between RGB and depth data features is traded off by using preferential separation followed by fusion of RGB and depth data streams, thus weakening the impact of the semantic segmentation results of 2D images caused by camera parameters and room illumination that cannot be controlled by camera parameter changes and room illumination. This reduces the impact of data occlusion, incompleteness, disorder, difficulty in feature extraction, and large data volume on semantic segmentation, thus improving the accuracy of semantic segmentation.

Due to various reasons, there are still some shortcomings and areas that need to be improved. According to some characteristics of the algorithm and experimental aspects of this article, the author believes that the method can be improved and expanded in the future from the following aspects.

1. Further complication of experimental data. The experimental data sources in this article are not enough, and more complex and more training data are needed so that the trained model can be more accurate for experiments.
2. The U-Net model will be optimized in the future to improve the speed and accuracy of its model, which will better achieve the purpose of this study.

## Data availability statement

The original contributions presented in the study are included in the article/supplementary material, further inquiries can be directed to the corresponding author/s.

## Author contributions

Writing—review and editing and supervision: WL. Resources and validation: SC. Supervision: JG. Validation and investigation: MJ. Visualization and investigation: XZ. Data curation and writing—original draft: ZW. Conceptualization, methodology, and software: JW. All authors contributed to the article and approved the submitted version.

## Conflict of interest

The authors declare that the research was conducted in the absence of any commercial or financial relationships that could be construed as a potential conflict of interest.

## Publisher's note

All claims expressed in this article are solely those of the authors and do not necessarily represent those of their affiliated organizations, or those of the publisher, the editors and the reviewers. Any product that may be evaluated in this article, or claim that may be made by its manufacturer, is not guaranteed or endorsed by the publisher.
